# The Needs and Experiences of Patients on Pain Education and the Clinical Reasoning of Physical Therapists Regarding Cancer-Related Pain. A Qualitative Study

**DOI:** 10.3389/fpain.2021.675302

**Published:** 2021-12-09

**Authors:** Tessa Eisen, Eline Menje Kooijstra, Ruud Groeneweg, Michelle Verseveld, Janine Hidding

**Affiliations:** Avans+, Master Oncologic Physical Therapy, University of Applied Sciences, Breda, Netherlands

**Keywords:** cancer, needs, experiences, pain, education, physical therapist, clinical reasoning, biopsychosocial

## Abstract

**Objective:** This study offers direction for interaction between physical therapists and patients about cancer-related pain during physical training. The study may increase awareness of rehabilitation strategies for cancer-related pain during and after cancer treatment.

**Methods:** Qualitative study, evaluating results of two qualitative studies. Data has been collected using semi-structured interviews, in which topics were discussed with patients and physical therapists. Respondents were adult patients with cancer in the Northern Netherlands with moderate to severe pain who followed physical training with a (oncologic) physical therapist. The physical therapists were respondents specialized in oncology and working with patients with cancer in a primary care setting in in the Netherlands. Data were analyzed using thematic analysis.

**Results:** Eighteen patients and fifteen physical therapists were interviewed. Data was categorized in statements regarding “patients' needs”, “patients' experiences” and “clinical reasoning of the physical therapist”. “Patients' needs” for education were personal and included needs for information about the cause, course and effect of pain in relation to cancer and/or medical treatment, needs for practical tools for reducing pain, needs for strategies dealing with pain in daily activities, and needs for information about additional treatment and care options. When discussing ‘patients’ experiences', patients mentioned that physical therapists are cautious to express their expectations of the progress of pain and to offer pain education with respect to the cause of pain, dealing with pain and limitations in daily life, exercising, posture, learning self-care and information about additional treatment and care options in cancer-related pain. Patients provided insight into their educational, mental, and social support relative to experiences with physical therapists. Additionally, when discussing the communication they experienced with physical therapists, patients used descriptors such as accessibility, empathy, trust, knowledge and eliminating uncertainties. Interviews with physical therapists regarding their clinical reasoning process in cancer-related pain described that they identified pain from anamnesis (medical history review) and performed screening and analysis for pain secondary to cancer (treatment), as type of pain and pain influencing factors. Thoughts and experiences about pain, the use of pain clinometry, the establishment of objectives and interventions for physical therapy and multidisciplinary treatment of cancer-related pain were also described.

**Conclusion:** Patients with cancer-related pain during physical training have personal needs regarding pain education and experience that specialized oncologic physical therapists focus on patient-centered information and self-management support. Specialized oncologic physical therapists analyze pain in the anamnesis and keep in mind the origin and impact of pain for the patient during screening and treatment. Different methods of pain management are used. It is recommended that physical therapists who give physical training take the initiative to repeatedly discuss pain.

## Introduction

Pain is a common symptom in patients with cancer. Due to an ageing population, the worldwide incidence of cancer will increase in the coming years (1). As the prevalence of patients with cancer is increasing as well, more people suffer from physical and psychological impairments caused by cancer or its treatment (2). In this study, cancer-related pain is defined as pain during or after cancer due to the cancer or its treatment. Cancer-related pain may be caused by the primary tumor or metastases that infiltrate, erode or inflame bone, viscera or nerves (3). The pain may also be caused by tissue or nerve damage due to cancer treatment (surgery, chemotherapy, radiotherapy, medication) (3). Moreover, patients with cancer may experience pain due to other diseases or physical symptoms (3).

In patients with cancer, the prevalence of pain fluctuates from 39% after curative treatment, 55% during cancer treatment and 66% in the end-of-life-phase (2). Moderate to severe pain [Numeric Pain Rating Scale (NPRS) > 5 (0–10)] is reported in 38% of the patients with cancer (2). Higher pain scores are associated with decreased physical activity (4, 5) and hamper activities of daily functioning (6). Cancer-related pain often has a chronic character: between 33% and 40% of the patients report pain over longer periods after cancer treatment (3). Cancer-related pain can be a hindrance to recovery and regaining functional levels as before diagnosis and has a negative impact on quality of life and social and emotional well-being (7). Persistent pain hinders return to work in patients with cancer (7). Over one third of the patients with cancer describe their pain as unacceptable or distressing (8).

In addition to physical causes of pain, psychological, social, and spiritual factors play a role in pain perception, as described in Saunders' Total pain model (9). The biopsychosocial model recognizes that a person's experience of pain is influenced not only by the degree of tissue damage but also by psychological and social factors (10). For cancer survivors proper identification of the nature of pain and accurate diagnosis and classification of pain is assumed to be important to achieve optimal pain management, which results in more adequate pain treatment (3). Despite significant progress in the knowledge and treatment of cancer-related pain in recent years, there are still large numbers of patients whose pain is poorly controlled or under-treated (3, 10). The literature states that there is a lack of knowledge among healthcare professionals regarding the assessment and treatment of pain during and after cancer treatment (2).

The rehabilitation of patients with cancer involves a multidisciplinary and biopsychosocial approach aimed at optimizing functioning, wellbeing and participation of cancer survivors in general and pain management specifically (11). The physical therapist plays an important role at all levels of cancer care (inpatient vs. outpatient) (11). Rehabilitation modalities of the physical therapist for pain during and following cancer treatment consist of exercises therapy, manual techniques and educational interventions to restore physical functioning (11).

For patients with chronic pain there is compelling evidence that pain education can have a positive effect on pain intensity, perceived disability, catastrophism and physical performance (12). Pain education with focus on understanding the neurophysiology of pain, can reduce incorrect thoughts and attitudes on pain and results in changes in physical activity, thereby realizing an active lifestyle (13, 14). Interventions on behavioral change are challenging (14) and patient-centered pain education is probably useful for the knowledge about pain of patients with cancer and may have a pain-reducing effect (15, 16). Promoting autonomy and control of pain in patients with cancer and the people around them, may contribute to the success of pain treatment (17). Due to heterogeneity in studies, the timing, content and frequency of offering pain education is unclear (15, 18). It is recommended to adapt the information to the level of knowledge and education of the patient, his physical and mental condition and the type of pain treatment (17). In order to provide pain education, patient orientation seems important (18).

Until now, the needs and experiences regarding pain education of patients with cancer-related pain during physical training in the Netherlands are unclear. Also, the clinical reasoning strategies of the specialized oncologic physical therapists behind their evaluation and treatment of cancer-related pain have to our knowledge not been published yet.

Understanding of the needs and experiences of patients with cancer-related pain during physical training and the clinical reasoning process of the specialized oncologic physical therapist regarding pain can result in better communication about pain and a more effective treatment. The purpose of this study is to answer the following questions: (1) What are the needs and experiences regarding pain education of patients with moderate or severe (NPRS > 5) cancer-related pain in the Northern Netherlands, during physical therapy treatment with a physical therapist? and (2) What is the clinical reasoning process of oncologic physical therapists regarding patients with pain during and after cancer treatment in the Netherlands?

## Materials and Methods

### Study Design

A qualitative study, using thematic analysis, was designed to get insight into the needs and experiences of patients with cancer-related pain during physical training and the clinical reasoning process of oncologic physical therapists regarding patients with cancer-related pain. In two qualitative studies, individual semi-structured in-depth interviews were conducted. Interviews with patients took place, first, from October 2017 to January 2018, at a patient's home or at an agreed location. Secondly, the interviews with physical therapists took place from December 2019 until March 2020 by video calls using the program Skype [Skype Technologies (Microsoft), Luxembourg].

### Study Population

The study population consisted of two different independent groups: a patient group and a physical therapist group.

The patients were respondents with moderate to severe cancer-related pain (NPRS > 5) due to the tumor or medical cancer treatment, receiving physical training by a physical therapist in the Northern part of the Netherlands. The following inclusion criteria were used: diagnosed with cancer in the curative or palliative phase, participated in physical therapy training for at least 6 weeks, moderate to severe pain at start of the training or during the training (NPRS > 5), good understanding of the Dutch language. Patients under 18 years of age, patients in end-of-life stage and patients with cognitive and physiological disorders were excluded. All physical therapists in the Northern Netherlands, who were members of the personal network of the researcher and the “Dutch Association of Lymphology and Oncology”, including “OncoNet”, were approached by email and informed about the study (19). Patients were recruited by their physical therapists. They received written information about the study and contact details of the researcher. Patients were included in order of registration.

The physical therapists were respondents in the Netherlands. The inclusion criteria were: a Master of Science degree in oncologic physical therapy or attending the graduation year of a Dutch oncologic physical therapy master's program, currently working with patients with cancer in a primary care setting and a good understanding of the Dutch language. Physical therapists were selected using a targeted sample. This was done taking into account enough variation in the characteristics of the participants: years of work experience, field of work and work setting. The oncologic physical therapists were recruited through the personal network of the researchers and the “Dutch Association of Lymphology and Oncology”, including “OncoNet” (19). Physical therapists were approached by email and informed about the study.

All respondents (patients/physical therapists) signed informed consent forms and provided sociodemographic data. When a respondent did not want to participate, the reason was described anonymously. Respondents were included until saturation occurred in the analysis.

Formal ethical approval for the interviews with patients was waved by the Medical Ethical Committee of the University Medical Center Groningen. The study was registered under number 201700600.

### Data Collection

Topics related to patients disease perception and self-management and to the clinical reasoning process of physical therapists on diagnosis and therapy related to pain during or after cancer treatment were identified in scientific literature (10, 11, 17, 20–27). These topics were discussed with an independent steering-board of physical therapists and a nurse. Two interview guides were developed. Patients were interviewed about information on pain, instructions on how to reduce or manage pain, education aimed at coping with pain and counseling and emotional support regarding pain during physical training. Therapists were asked to describe their diagnostic process regarding patients with cancer-related pain in relation to functions, activities, participation, personal and external factors. They were also asked to describe their therapeutic process and how clinical reasoning contributed to their treatment plan and treatment goals regarding patients with cancer-related pain. In both groups pilot interviews were conducted. After reflection and consensus on the adaptations, the preliminary interview guide was adjusted.

The interviews were recorded using a Dictaphone. The audio materials were transcribed into text. During the interviews, the researchers made notes of remarkable statements and of factors that might cause bias or were of interest regarding the study question. A member check was used, giving the respondents the opportunity to assess the correctness of the text and give feedback.

### Data Analysis

Contact details and sociodemographic data were pseudonymized and stored separately from the interview. All files were stored in a secured safe, and will be kept for 10 years. Personal patient information and audio recording were deleted after a member check of the transcript. Descriptive analyses were used to describe characteristics of the respondents.

Patient data were processed using Kwalitan (version 7.0) (28). Physical therapist data were processed using Microsoft Word 2018. The first three interviews of both study arms were analyzed independently by the main researchers (TE, EMK) and peer reviewers (TEK, ZM). Fragments relevant to the study question were selected from the transcript and open codes were discussed, after which consensus was achieved. The open codes were gathered into categories from which themes were identified. This was organized into a tree structure. During the coding process a log was posted.

Based on the interviews, new topics and insights in the study question were discussed in the steering-board and added to the interview guides. Data collection and data analysis alternated continuously in an iterative process, each time looking at how the analysis results contributed to answering the study question. Based on this process, topics were added to the interview guide during the process.

## Results

Twenty patients were enrolled. Eighteen patients were included and two patients did not meet inclusion criteria. Fifteen female patients and three male patients participated in the study. Twelve patients were treated curatively and six patients had an incurable form of cancer. The mean age of the patients was 62 years with a range of 36 to 75 years. Due to anxiety about a telephone consultation with the physician, the researcher ended one interview earlier. Fifteen physical therapists were included, of whom fourteen were female and one was male. The mean age of the physical therapists was 34.3 years (SD 8.6). The characteristics of the patients and physical therapists are shown in Table 1. The mean duration of interview time for the patients was 43 mins and for the physical therapists 57 mins.

**Table 1:** Characteristics respondents.

**Variable**	Patients^*^	Physical therapists^*^
**Number**	18	15
**Age in years** [average (range) (SD)]	62 (range 39)	34.3 (SD 8.6)
**Genus**		
Woman	15 (83%)	14 (93%)
Man	3 (17%)	1 (7%)
**Living area**		
Northern Netherlands	18 (100%)	12 (80%)
Central Netherlands		3 (20%)
Southern Netherlands		0
**Work experience in years** [average (SD)]		11 (SD 7.2)
**Study physical therapists**		
- MSc oncologic physical therapy		9 (60%)
- MSc oncologic physical therapy graduation year		6 (40%)
**Additional training on pain**		3 (20%)
**Education level of the patients**		
Basic and secondary education	3 (17%)	
Lower vocational education	2 (11%)	
Secondary vocational education	9 (50%)	
Higher vocational education	3 (17%)	
Scientific education	1 (6%)	
**Diagnose**		
Mammary carcinoma	12 (67%)	All forms in ratio of prevalence were seen in practice
Prostate carcinoma	3 (17%)	
Ovarian carcinoma	2 (11%)	
Desmoid tumor-aggressive fibromatosis	1 (6%)	
**Medical treatment design**		
Curative	12 (67%)	Curative, palliative, terminal
Palliative	6 (33%)	
**Medical treatments**		
Surgery	16 (89%)	
Chemotherapy	14 (78%)	
Radiotherapy	13 (72%)	
Anti-hormonal therapy	8 (44%)	
Immunotherapy	4 (22%)	

* *Values given in number, if different, this is indicated behind the variable*.

^*^Percentages are rounded.

^*^SD, standard deviation, MSc, Master of Science.

**Figure 1:**
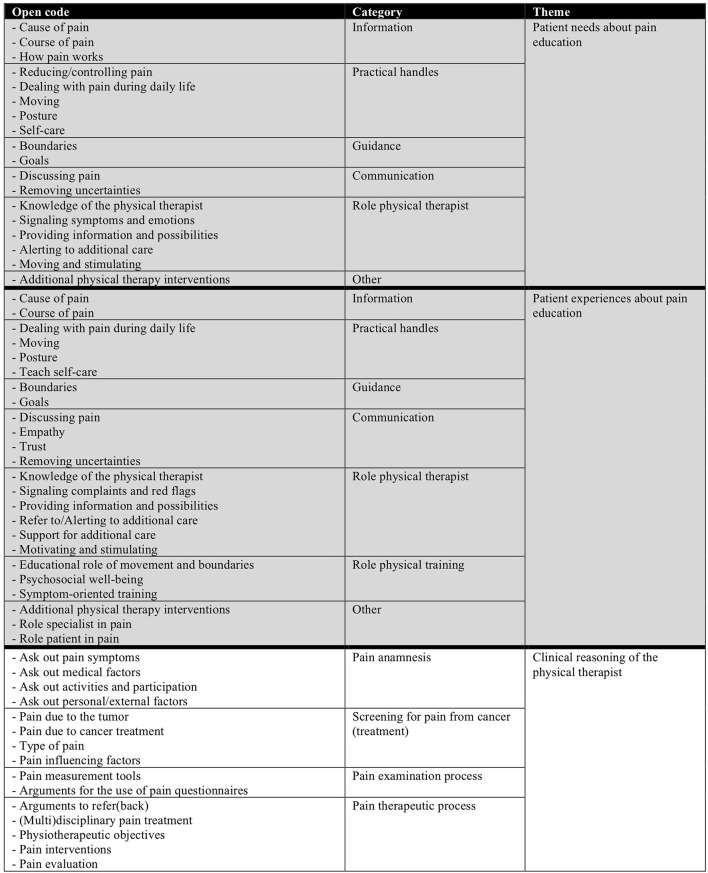
Code tree - The needs and experiences of patients on pain education and the clinical reasoning of physical therapists regarding cancer related pain.

Thematic analysis resulted in three themes: patients' needs, patients' experiences, and clinical reasoning of the physical therapist, see code tree, Figure 1.

### Patients' Needs

Patients participated in physical training, when necessary using pain relief (through medication, radiotherapy or surgery), to improve or maintain muscle strength, cardio- respiratory fitness, balance or range of motion. During physical training, patients had questions about pain related to cancer and medical treatment. Patients indicated a need for information on the cause and course of pain. There were patients who wanted to know how pain works. About the need for information was said:

*“You don't know if the pain is part of the cancer treatment or not. You also feel very insecure in terms of the pain.” (Patient 13)*

Patients in the palliative phase wanted information about the current pain, but did not want to look too far ahead towards possible “longer-term pain”. There were patients in the palliative phase who indicated they felt a need for information about psychological care and relaxation exercises related to pain, which had not yet been discussed with the physical therapist.

Patients needed information about additional treatment and care options in cancer-related pain. Patients advocated for themselves and took responsibility in asking questions and looking for information. On the other hand, when patients were unfamiliar with complementary treatment options, they didn't know what to ask for. One patient said:

*“In all medical treatments and around it, you are helped. But actually, you're on your own after that. If you ask specifically, you'll get an answer. But if you don't know what to ask for, it remains unclear.” (Patient 11)*

It was indicated that pain limited daily activities and patients had to deal with the perceived limitations. Patients said they needed practical tools to reduce and deal with pain during daily life:

*“What can I do and can't do? How can I deal with pain myself in terms of reducing it?” (Patient 16)*

Patients needed an open dialogue to discuss pain and appointed questions by the physical therapist as desired. One patient said:

*“That's what I expect. Otherwise, I'd feel a little bit like I'd have to figure it all out on my own.” (Patient 5)*

Needs for information, instructions or guidance differed among the patients. There were patients who were adequately informed by the doctor or oncologic nurse, others stated to have gathered information on their own or that new additional information might become “too much.”

### Patients' Experiences

Patients experienced that the physical therapist discussed pain, answered questions about pain related to cancer and were cautious about statements regarding the course of pain. Physical therapists offered information about additional treatment and care options and encouraged discussions on pain with other healthcare providers. A patient said:

*“They offer you all kinds of things. Why don't you go talk about this? I think she gives me a lot of support.” (Patient 1)*

It was mentioned that the physical therapist matched problems in daily life with instructions regarding daily activities, workload and resilience, daily schedule, work, posture instructions or recommendations regarding “exercise despite pain”. In addition, patients experienced that home exercise schedules, self-massage techniques, breathing and relaxation exercises, and conscious exercise behavior were part of self-care on pain. It was indicated:

*“There are all kinds of tips. You'd better do this. You'd better do it this way. If you divide your energy a little, you also suffer less from the pain.” (Patient 13)*

By information or instructions patients identified the educational role of physical training in recognizing boundaries and importance of continuing of moving with pain, experiencing mental and social support. About the role of physical training was said:

*“You have to persevere from time to time and then just feel the pain. If it's over after an hour, it's not all that bad. I've also learned to do it dosed, not to go on like crazy. I have to move because it is good for me, building up.” (Patient 5)*

With regard to accepting and dealing with limitations due to pain, the physical therapist discussed, supervised and showed understanding. About boundaries regarding activities and adjusting goals in case of pain, a patient said:

*“You get it unsolicited. Because of your conversations together, that'll come back to you. Why do I have to walk those seven kilometers? You can walk five kilometers. Then you will automatically adjust your goals.” (Patient 12)*

Commonly recurring in the interviews were answers like discussing, asking and telling your story. Regarding communication with the physical therapist about pain, patients experienced accessibility, knowledge, empathy, a relationship of trust, and reassurance together with discussing pain.

*“It is very accessible. You can ask all the questions, nothing is weird, no matter what crazy pains. Actually, they've been telling me a lot. What are some side effects and what I can possibly do about them?” (Patient 17)*

In addition to physical training, the physical therapists offered additional treatments to relieve pain, such as complaint specific and functional exercise therapy, stretching exercises, massage, breathing and relaxation exercises, scar treatment, medical taping, lymphedema treatment, pelvic floor training, manual therapy, and ultrasound examination.

Patients were also alerted by physical therapists to additional care aimed for pain, such as psychologists, occupational therapists, hyperbaric oxygen therapy, medical pedicures, additional diagnostics or ergonomic support tools, and yoga.

### Clinical Reasoning of the Physical Therapist

The physical therapists mentioned that during the anamnesis they asked for pain symptoms and pain course, for example: pain location(s), duration of pain sensations, pain description and pain score on the NPRS. They inventoried medical factors as the cancer (treatment), previous medical interventions, pain history, (pain) medication and co-morbidities. In addition, physical therapists mentioned that they asked for restrictions in activities and participation due to pain and pain relieving/exacerbating activities. The therapists also mentioned that they asked about the personal factors, as reason for physical therapy consultation and expectations, mental state, lifestyle, self-management strategies, pain coping/behavior, pain/illness perception and influence of home, work, social or health care providers on pain perception, and pain behavior.

Physical therapists indicated that they screen for pain caused by cancer (tumor, metastatic disease, lesions, breakthrough pain, paraneoplastic syndrome) or its treatment (radiotherapy, surgery, anti-hormonal treatment, immunotherapy, chemotherapy). One physical therapist said:

*“As a physical therapist we try to be alert on that of course. For example, if somebody doesn't have neuropathy, but is at risk, we will ask the patient every time if there are any symptoms.” (Physical therapist 1)*

It was mentioned that the physical therapists screen for the type of pain (nociceptive or neuropathic pain). A number of physical therapists indicated that they also screen for central sensitization, while others did not. In addition, to all physical therapists screening for factors influencing pain as physical activity in relation to pain behavior, emotions, social and somatic factors, and cognitions were important.

The physical therapists assessed pain by inspection of the affected region and position of the body part with pain. Furthermore, palpation, examination regarding movement, muscle and nerve stretching tests, muscle strength tests and cardio-pulmonary capacity tests were performed. They also assessed aspects of pain using a pain/symptom diary.

Pain and pain influencing factors in relation to activities and participation and personal factors (experienced health or complaints, fatigue, depression, quality of life, pain coping, kinesiophobia, pain catastrophizing) were assessed. Table 2 shows an overview of the mentioned measurement tools.

**Table 2:** Measurement tools mentioned by physical therapists for measuring factors related to pain in cancer.

**Mentioned measurement tool**	**Measurement properties**
Numeric Pain Rating Scale (NRS) (6)	Subjective measure in with individuals rate their pain on a scale of zero to 10, 10 being the worst (6).
Central Sensitization Inventory (CSI) (29)	Self-report outcome measure designed to identify patients who have symptoms that may be related to central sensitisation (29).
Patient Specific Functional Scale (PSFS) (30)	Self-report outcome measure of function that could be used in patients with varying levels of independence (30).
Pain Disability Index (PDI) (31)	Measures the impact that pain has on the ability of a person to participate in essential life activities (31).
Four-Dimensional Symptom Questionnaire (4DSQ) (32)	Self-report questionnaire to assess distress, depression, anxiety and somatization (32).
Distress Thermometer and Problem List (33)	The thermometer measures distress in a similar way to pain on a scale of zero to 10, 10 being the worst. The Problem list allows patients to inform their health care provider if they are having concerns in areas such as practical, family, emotional, spiritual, and physical problems (33).
Visual Analogue Scale Fatigue (VAS-F) (34)	To evaluate fatigue severity on a scale of zero to 10, 10 being the worst (34).
Multidimensional Fatigue Inventory (MFI) (35)	A self-report instrument designed to measure fatigue. It covers the following dimensions: General Fatigue, Physical Fatigue, Mental Fatigue, Reduced Motivation and Reduced Activity (35).
Center for Epidemiological Studies Depression Scale (CES-D) (36)	Self-report measure of depressive symptoms (36).
European Organization for Research and Treatment for Cancer Quality of Life Questionnaire (EORTC) (37)	Assesses patients' health-related quality of life in oncology (37).
Pain Coping Inventory (PCI) (38)	Identifies the coping strategies of people with pain symptoms (38).
Pain Catastrophizing Scale (PCS) (39)	Self-report measure of catastrophizing in the context of actual or anticipated pain (39).
Tampa Scale for Kinesiophobia (TSK) (40)	Measures fear of movement or re (injury) as linked to thoughts and beliefs about pain (40).

Clinical reasoning with the use of pain clinometry in relation to personal factors was varied. Some physical therapists indicated that they received enough information from their interview techniques during the intake. Other physical therapists indicated that this clinometry could be used in relation to the patients' needs, as guidance for a conversation or to provide insight. One physical therapist said:

*“When you think now this patient catastrophizes a lot, which of course can amplify the pain, that you examine this with a questionnaire and use it as a guidance for a good conversation.” (Physical therapist 3)*

It was mentioned by the physical therapists that they advise patients with pain symptoms to contact a clinician when the pain symptoms are beyond the professional competencies of the oncologic physical therapist, when the patient is in need or in doubt, when there is unexplained pain or an abnormal pain course. In pain treatment, physical therapists collaborate with psychologists, social workers, occupational therapists, pain clinics, specialists in return to work, hyperbaric medicine, and complementary medicine. In relation to goal settings in physical therapy treatment, physical therapists stated that it is important to create realistic expectations, that goals are formulated SMART (Specific, Measurable, Attainable, Relevant, Time-bound) and that the treatment phase and patients' needs should be taken into account.

The following objectives in pain treatment were mentioned by the physical therapists: improvement of function and activity level, education to cope with the pain and encouragement of self-management and behavioral change to cope with pain. The physical therapists stated that pain treatment is often not the main goal, but pain management is important to achieve goals on activity and participation level. If goals were not met by physical therapy alone, multidisciplinary care would be considered.

One of the interventions mentioned to relief pain was pain education. The following issues were discussed when giving pain education: consequences of cancer treatment, functioning of the pain system and impact of medication, relation between pain and coping style, role of emotions, workload and resilience, lifestyle, and behavior. There were physical therapists who involved close family and friends in the treatment process. For some physical therapists it was difficult to provide education about pain. They did not feel comfortable or competent and preferred to leave education about pain to other healthcare disciplines. One physical therapist said:

*“What do I tell and what not and in what way do I convey my message well?” (Physical therapist 9)*

The use of general and specialized exercise therapy as a pain intervention (muscle strength training, mobility or aerobic and resistance training) and the use of graded activity or graded exposure were mentioned. Physical therapists used exercise therapy to promote movement strategies, body awareness, self-confidence and anxiety reduction. Also other interventions complementary to exercise therapy were mentioned: hands-on muscle stretching/connective tissue and joint mobilization techniques, kinesiotape and compression garments as well as relaxation therapy, hydrotherapy, heat or cold therapy and Transcutaneous Electrical Neurostimulation. Self-management was encouraged by giving the patient exercise schedules for at home or teaching the patient or partner self-management techniques.

## Discussion

In this qualitative research, patients diagnosed with cancer, experiencing moderate to severe pain, indicated their needs and discussed their experiences regarding pain in relation to physical therapy treatment and oncologic physical therapists discussed their clinical reasoning process regarding patients with pain during and after cancer treatment.

Patients need pain education concerning the cause, course, and effect of pain in relation to cancer and/or the medical treatment. They need practical tools for reducing pain, dealing with pain in daily activities and information about additional treatment and care options. Patients experience that physical therapists are cautious in expressing their expectations of the development of the pain. Their physical therapists offer pain education about the cause of pain, dealing with pain and limits in daily life, training, posture, learning self-care strategies and information about additional treatment, and care options in cancer-related pain. Patients experience educational, mental and social support in relation to physical training. Communication with the physical therapist about pain is mentioned in connection with accessibility, empathy, trust, knowledge, and removing uncertainties.

Oncologic physical therapists inventory the pain, including the location, characteristics, mechanisms, expression, and function of the pain. They assess pain in relation to functional limitations, psychosocial factors, and current analgesic treatment. The physical therapists use interviewing techniques and clinometry to support the clinical reasoning process for patients' beliefs regarding pain, coping mechanisms and self-efficacy. The treatment is shaped on personal preferences and individual needs. Although the main role of the physical therapist is to restore physical function, interventions on understanding pain mechanisms and coaching on pain behavior and additional interventions to relieve pain are also implemented in the treatment.

Based on the findings of this study, the researchers expect that the identification of personal needs of patients with cancer-related pain during physical training contributes to patient- oriented treatment, offering education, and self-management support, whereby a connection must be made with the person and the life he or she wants to lead (41). This perspective can offer the patients autonomy to make their own choices for tailored care and to participate actively in their own care process. The information needs of patients about cancer-related pain or medical treatment correspond to disease perceptions, as described in Leventhals “Common sense model of selfregulation” (20). Disease perceptions give direction to the way patients deal with complaints in daily life and are therefore important for self-management (20). Patients indicate to need practical tools to reduce pain and deal with pain in daily life. Proper pain management is important, in order to be able to live “normally” and perform daily activities (42). The pain education, mentioned by patients in this study, seems to support self-management in cancer-related pain (22). The results of the interviews with physical therapists also show that they give pain education and stimulate self-management.

Cancer-related pain is influenced by many factors, such as a patients' previous experience with pain or the patients' attitude towards pain and use of analgesics (43). To build a good relationship with the patient and address underlying patient-related barriers to adequate pain management, these factors have to be understood by the physical therapists and need careful examination (43). The results of this study show that the oncologic physical therapists examine physical limitations and psychosocial factors in relation to pain and use clinometry for that purpose. Measurement instruments which assess not only pain, but also experiences regarding physical and emotional well-being, can support the holistic approach of patients with cancer pain (11, 25, 44–46). The choice whether or not to use measurement instruments for pain, as well as the choice which measurement instruments, varies among the physical therapists. This variation can be explained by a lack of guidelines regarding pain assessment.

Physical therapists are recommended to give patients with cancer-related pain, education based on pain (neuro) science with a biopsychosocial approach to remove barriers to rehabilitation and to promote adequate pain behavior and cognitions (47). Why oncologic physical therapists find this skill difficult can possibly be explained by barriers of the physical therapists in the application of psychological interventions (48). Physical therapists experience barriers with regard to available time, knowledge, and their role in educational support (48). It cannot be deduced from the patients' experiences that information about neurophysiology was offered. The physical therapists' cautiousness in relation to the diversity of cancer-related pain may underlie this and requires a good classification of pain (24). In addition to the examination of physical limitations and psychosocial factors, physical therapists indicate that they identify the type of pain, which supports the clinical reasoning process and the choice of adequate pain interventions (3, 24, 49). This study shows that several physical therapists do not screen for central sensitization (CS). CS is a phenomenon that involves hypersensitivity of the central nervous system (24, 49). The reason why several physical therapist do not screen for CS can perhaps be explained because there is a debate concerning the terminology used to describe the clinical presentation of pain hypersensitivity when patients present with features of CS (29). Also, there are limited guidelines for the recognition of CS pain among cancer survivors (24). The “Central Sensitization Inventory” appears to be a valid and reliable tool to quantify symptoms of central sensitization and may support the clinical reasoning and treatment process (29).

This study indicates that patients during physical training find communication and a relationship of trust important when discussing pain. In the literature patient-oriented pain education is recommended, as where patients should be approached as a person with needs in physical, emotional, spiritual and relational dimensions (18, 41). It is expected that physical therapists communicate in an open communicative relationship with patients (41). Confidence in the healthcare provider is of significant importance for good communication and pain knowledge of patients with cancer (50). With less confidence, patients are more reluctant to express pain and are less likely to follow advice (50). Patients state that repeated contact with the physical therapist provides accessibility for asking questions, in which patients value oncological knowledge of the physical therapists.

Treatment of cancer and insight in multidimensional health problems require a broad-based area of knowledge of physical therapists and often patients with pain require multidisciplinary treatment (10, 17). Masters of Oncologic physical therapy are expected to search for scientific literature, critically assess it and use scientific knowledge when making choices regarding individual care (51). Multidisciplinary treatment and dissemination of knowledge to patients, colleagues and other healthcare professionals are responsibilities of the oncologic physical therapist (51). This study shows that the specialized physical therapist treats cancer-related pain multidisciplinary.

Findings in this study that indicate educational values in relation to physical training may be based on guiding and coaching skills of the physical therapist (51). No representative study has been found to verify these results or any relationship. The findings provide perspectives for further research on the effectiveness of pain education during physical training aimed at supporting self-management of cancer-related pain.

A strength of this study is the continuous peer debriefing process in both study groups: interview choices and data analysis were discussed with the peer-reviewers and a steering board. The member check of the transcripts also contributed to the validity of this study.

A positive aspect of this study is that patients are included with a wide range in age, education level and during different phases of treatment and disease. It is striking that many women with breast cancer participated in this study. This is partly explained by the incidence of breast cancer and because the Dutch “Mamma carcinoma Guideline” recommends discussing physical training with each patient (52, 53). In addition, being of “young age” and “female” are risk factors for postoperative chronic pain (7). The number of men and patients with other cancers is under- represented in the study. In addition, the population in the Northern Netherlands is not fully representative for the Netherlands and is different in terms of indigenous population and education level (54). It is known that, depending on the cultural background, differences in information regarding symptom management are experienced (55). As the demographic differences between the population of this study and the Dutch population are small, it is expected not to affect the generalizability of the patients' needs and experiences.

The study population of included oncologic physical therapists appears to be representative for the Netherlands: they work in primary care, they work in six different provinces of the Netherlands, there is a large standard deviation in age and work experience of the physical therapist and they see patients with varying cancer diagnoses in their practice. However, only one man was interviewed. The clinical reasoning process of physical therapists with a Master in Oncologic physical therapy may be different from those in general physical therapy practice, therefore results should not be generalized to all physical therapists. The study population of the oncologic physical therapists may not be fully representative for other countries, because there may be a difference in education and options to specialize as an oncology physical therapist.

The application procedure of patients respondents by physical therapists and physical therapist respondents by the researchers, the profession of the researchers (physical therapists) and the face-to-face interviews with patients cannot rule out that the results are subject to any selection bias or interviewer bias. To exclude confirmation bias, the transcripts of the patients interviews were reviewed by a nurse on open questioning. During the semi-structured interviews, respondents were given a lot of space to tell and explain. This may have some impact on the reproducibility of the study, but this provided a lot of insight into the needs and experiences of patients and the clinical reasoning of physical therapists regarding pain. The choice of conducting the physical therapists' interviews by video call was made because of time efficiency, as no travelling was necessary and respondents could choose by themselves where and when they wanted to be interviewed. This may have led to different interpretations of interview elements, due to less visibility of body language.

A follow-up study that includes more diversity in diagnoses of cancer, has insight in the type of pain of patients with cancer and conducted in other parts of the Netherlands or other countries may lead to more specific insights.

It is recommended that physical therapists, training patients with cancer, evaluate and discuss pain and its consequences in daily life and mental health repeatedly and involve other healthcare disciplines that could be helpful, to meet the needs of the patients with cancer-related pain. For optimal care, according to the principles of evidence-based practice, it is recommended for patients with cancer-related pain to consult an oncologic physical therapist with knowledge of cancer (treatment) and expertise in pain treatment.

In conclusion, patients with cancer-related pain during physical training have personal needs regarding pain education and experience that specialized physical therapists focus on patient-centered information and self-management support. Specialized oncologic physical therapists analyze pain in the anamnesis and keep in mind the origin and impact of pain for the patient during screening and treatment. Different methods of pain management are used.

## Data Availability Statement

The original contributions presented in the study are included in the article/supplementary material, further inquiries can be directed to the corresponding author.

## Ethics Statement

The study involving patient participants was reviewed and approved by Medical Ethical Committee of the University Medical Center Groningen. The study was registered under number 201700600. The patients/physical therapists provided their written informed consent to participate in this study.

## Author Contributions

TE and EK: conceptualization, writing, and original draft preparation. TE, EK, and JH: literature search and data extraction. RG, MV, and JH: writing, review & editing and supervision. All authors contributed to the article and approved the submitted version.

## Funding

The publication of this work is partially funded by the Dutch Association for Physical Therapy within Lymphology and Oncology (NVFL) and Avans+ University of Applied Sciences, Netherlands.

## Conflict of Interest

The authors declare that the research was conducted in the absence of any commercial or financial relationships that could be construed as a potential conflict of interest.

## Publisher's Note

All claims expressed in this article are solely those of the authors and do not necessarily represent those of their affiliated organizations, or those of the publisher, the editors and the reviewers. Any product that may be evaluated in this article, or claim that may be made by its manufacturer, is not guaranteed or endorsed by the publisher.
